# Association of Uncoupling Protein 1 (*UCP1*) gene polymorphism with obesity: a case-control study

**DOI:** 10.1186/s12881-018-0715-5

**Published:** 2018-11-20

**Authors:** Shahanas Chathoth, Mona H. Ismail, Chittibabu Vatte, Cyril Cyrus, Zhara Al Ali, Khandaker Ahtesham Ahmed, Sadananda Acharya, Aisha Mohammed Al Barqi, Amein Al Ali

**Affiliations:** 1 Department of Genetic Research, Institute for Research and Medical Consultation, Imam Abdulrahman Bin Faisal University, P.O. Box 1982, Dammam, 31441 Saudi Arabia; 2 Department of Internal Medicine, King Fahd Hospital of the University, Imam Abdulrahman Bin Faisal University, Al-Khobar, Saudi Arabia; 3 Department of Pathology, University of Alabama, Birmingham, USA; 4 Department of Public Health, College of Public Health, Imam Abdulrahman Bin Faisal University, Dammam, Saudi Arabia; 5 Department of Biochemistry, Imam Abdulrahman Bin Faisal University, Dammam, 31441 Saudi Arabia

**Keywords:** *UCP1*, *NCP1*, Moderate-obese, Extreme-obese, Diabetes, HDL, LDL, Cholesterol, Metabolic disorder

## Abstract

**Background:**

Obesity is one of the main causes of morbidity and mortality worldwide. More than 120 genes have been shown to be associated with obesity related phenotypes. The aim of this study was to determine the effect of selected genetic polymorphisms in Uncoupling protein 1 (*UCP1*) and Niemann-Pick C1 (*NPC1*) genes in an obese population in Saudi Arabia.

**Methods:**

The genotypes of rs1800592, rs10011540 and rs3811791 (*UCP1* gene) and rs1805081 and rs1805082 (*NPC1* gene) were determined in a total of 492 subjects using TaqMan chemistry by Real-time PCR. In addition, capillary sequencing assay was performed to identify two specific polymorphisms viz., rs45539933 (exon 2) and rs2270565 (exon 5) of *UCP1* gene.

**Results:**

A significant association of *UCP1* polymorphisms rs1800592 [OR, 1.52 (1.10–2.08); *p* = 0.009] was observed in the obese cohort after adjusting with age, sex and type 2 diabetes. Further BMI based stratification revealed that this association was inconsistent with both moderate and extreme obese cohort. A significant association of *UCP1* polymorphisms rs3811791 was observed only in the moderate-obese cohort [OR = 2.89 (1.33–6.25); *p* = 0.007] but not in the extreme-obese cohort indicating an overlying genetic complexity between moderate-obesity and extreme-obesity. The risk allele frequencies, which were higher in moderate-obese cohort, had abnormal HDL, LDL and triglyceride levels.

**Conclusion:**

The rs1800592 and rs3811791 of *UCP1* gene are associated with obesity in general and in the moderate-obese group in particular. The associated *UCP1* polymorphisms in the moderate-obese group may regulate the impaired energy metabolism which plays a significant role in the initial stages of obesity.

**Electronic supplementary material:**

The online version of this article (10.1186/s12881-018-0715-5) contains supplementary material, which is available to authorized users.

## Background

Obesity represent a serious public health problem worldwide and is associated with co-existing diseases, including cardiovascular diseases, type 2 diabetes mellitus (T2DM), musculoskeletal conditions and various cancers [1–3]. The prevalence of obesity in a population is an indicator of its health status and in recent years obesity has reached epidemic proportions. In the last few decades, Saudi Arabia has witnessed an increased prevalence of obesity [4]. A recent survey revealed that overall, 28.7% of 10,735 Saudi nationals recruited for a Saudi Health Information Survey in 2013 were obese, with a higher prevalence in females than males (33.5% vs 24.1%) [5].

Obesity and weight gain have been reported to be associated with several genes in addition to known factors, such as diet and lack of exercise [6]. It has also been reported that obesity is influenced by genetic variations and ethnicity [7, 8]. The polymorphisms which are involved in obesity were shown to be discordant in their association in various e ethnic populations. Therefore, genetic variations in ethnic populations need to be determined to validate the genetic significance of the polymorphisms. The majority of the genetic variations in obesity are related to the genes associated with energy metabolism. Uncoupling proteins are associated with the pathogenesis of obesity and T2DM by deregulation of energy expenditure, thermogenesis and reduction in oxidative stress [9]. Several studies have reported that polymorphisms of the *UCP1* gene such as, g.-3826A > G (rs1800592), g.-1766A > G (rs10011540) and g.-112A > C (rs3811791) in the promoter region, and p.Ala64Thr (rs45539933) and p.Met299Leu (rs2270565) in the codon region are associated with obesity and T2DM [10–15]. The Niemann-Pick C1 gene (*NPC1*), another reported genetic determinant of obesity, is a gene for transmembrane glycoprotein located in the limiting membrane of late endosome/lysosome (LE/LY) and mediates intracellular trafficking of sterols [16–18]. It has been reported that rs1805081 (p.His215Arg) and rs1805082 (p.Ile858Val) polymorphisms of the *NPC1* gene are associated with early-onset and morbid adult obesity in a European population and Chinese children [19–21]. A Genome Wide Association Study conducted on Mexican children found a significant association of risk allele *NPC1* rs1805081 with increased fasting glucose levels and decreased fasting serum insulin levels [22].

The *UCP1* and *NPC1* genes are known to be involved in the regulation of energy metabolism and the role of the polymorphisms in these genes with respect to obesity is arguable due to the diverse results of studies performed in different ethnicities. The aim of this study is to determine the association of the polymorphisms of the *UCP1* [rs1800592, rs10011540, rs3811791, rs45539933 and rs2270565] and *NPC1* [rs1805081 and rs1805082] genes in a Saudi population.

## Materials and methods

### Subjects eligibility and recruitment

A total of 337 obese patients and 155 non-obese control subjects attending King Fahd Hospital of the University were included in the study. All patients and controls were Saudi origin. The inclusion criteria for obese patients included BMI ≥30 kg/m^2^ and age between 18 and 60 years. The control group comprised healthy subjects with a BMI < 30 kg/m^2^. The patient cohort was grouped as moderate-obese and extreme-obese based on the heterogeneity of variations in suspected etiology, prevalence, mortality rate and anthropometric measures, mainly BMI. The moderate-obese cohort comprised patients with a BMI ≥30–39.9 kg/m^2^ and the extreme-obese cohort with a BMI ≥40 kg/m^2^. These cohorts were further subdivided based on age, gender, abnormal biochemical parameters and co-existing conditions. Written informed consent was obtained from all participants. The study was approved by Institutional Review Board of Imam Abdulrahman Bin Faisal University of Dammam (IRB-2013-01-008).

### Sample collection and biochemical parameters estimation

Five mL of whole blood was collected in EDTA anti-coagulated vacutainers from patient and control subjects after an overnight fast. Biochemical parameters, including total cholesterol, high-density lipoprotein (HDL), low-density lipoprotein (LDL), triglycerides levels, fasting blood glucose (FBG) level and insulin levels were determined at King Fahd Hospital of the University using Siemens Dimension RxL chemistry system (Siemens, Erlangen, Germany) and other details of co-existing medical conditions were collected from the hospital medical records.

### Mutation detection by TaqMan SNP genotyping assay

Genomic DNA was isolated using Promega DNA isolation kit (Promega, Madison, USA) according to the manufacturer’s instructions. Concentration and purity of isolated DNA were determined using Nanodrop spectrophotometer and then stored at − 20 °C until the day of mutation analysis. TaqMan chemistry based Real-Time PCR method was used to detect the SNPs of *UCP1* (rs1800592, rs10011540 and rs3811791) and *NPC1* (rs1805081 and rs1805082). TaqMan probes were synthesized by Applied Bio systems, (Thermo Scientific, CA, USA) which detect both wild and mutant alleles. The assay was conducted as per the manufacturer’s instructions. ABI 7500 fast real-time PCR system proprietary software (Thermo Scientific, CA, USA) was used for analysis and interpretation of the results.

### Mutation detection by capillary sequencing assay

Distribution of polymorphisms and novel mutations on exon 2 and exon 5 of *UCP1* gene was carried out using capillary sequencing by ABI 3500 genetic analyzer (Thermo Scientific, CA, USA) as previously reported [23]. The targeted gene sequence was amplified using polymerase chain reaction with specific primers for Exon 2 (Fwd-5’TCTGCACCTTTCTTATTTC3’ Rev-5’TCTCGCCAATTTGTTATGAA3’) and Exon 5 (Fwd-5’CAAAAGTCTGATGTTGAC3’ Rev-5’GAAATCTGTGGCAAGGAAAAGT3’) on a thermal cycler S1000 (Bio Rad, Hercules, California, USA). BigDye Direct sequencing master mix was used to perform the cycle sequencing reaction. The Sequencing Install Standard and BigDye® Terminator v3.1 Kit. POP7 polymer and 50 cm capillary (Thermo Scientific, CA, USA) were used in this procedure. 10 μl of purified product was loaded in 96 well plates and analyzed using ABI genetic analyzer 3500 (Thermo Scientific, CA, USA) for sequence detection.

The DNA sequence was then viewed on sequence analysis software. Sequence alignment was performed using the NCBI alignment and codon code analyzer software with reference sequence of *UCP1* gene (NG_012139.1).

### Statistical analysis

Collected data were summarized as mean ± SD. The patient and control demographic parameters, including biochemical and clinical data, were tested for statistical difference using students’ “*t”* test for continuous variables and Chi-square test for discrete variables with one degree of freedom. Risk allele frequencies (RAF) were estimated by direct counting of the test allele divided by the total number of alleles. Multiple variable logistic regression model using age, sex, and absence/presence of T2D as covariates was performed to assess the association of these SNPs with obesity. The *p* value < 0.0125 has been considered as significant for regression analysis as per Bonferroni-correction. All statistical analyses were performed using SPSS software (version19) and GraphPad Prism 7.03.

## Results

### Clinical, biochemical and genotypic characteristics of the Unstratified case and control subjects

A total of 337 obese patients (Male = 138, Female = 199) with a mean BMI of 39.59 ± 10.32 kg/m^2^ and a mean age of 47.41 ± 12.79 years were included in this study. The control population included 155 healthy volunteers (Male = 76, Female = 79) with a mean BMI of 24.09 ± 2.6 and a mean age of 43.86 ± 14.54 years. Of the 337 obese patients, 235 were T2DM patients and 85 had hypertension (HTN). The levels of FBG, triglycerides, and HDL were significantly different (*p* < 0.05) between the patient and control groups. The clinical and biochemical parameters of the patients and controls are presented in Table 1.

**Table 1:** Clinical and biochemical parameters of the study cohort

Clinical and biochemical parameters	Control (*n* = 155) (mean ± SD)	Patient (*n* = 337) (mean ± SD)	*p*-value
Age (years)	43.86 ± 14.54	47.41 ± 12.79	**0.006**
Male / Female, *n* (%)	76 (49) / 79 (51)	138 (41) / 199 (59)	0.097
BMI (kg/m^2^)	24.09 ± 2.6	39.59 ± 10.32	**< 0.005**
FBG (mg/dL)	120.58 ± 56.75	152.08 ± 71.66	**< 0.005**
Triglycerides (mg/dL)	100.00 ± 62.45	136.85 ± 78.48	**< 0.005**
LDL (mg/dL)	115.25 ± 42.90	111.59 ± 36.56	0.331
Cholesterol (mg/dL)	189.34 ± 134.62	179.58 ± 40.89	0.225
HDL (mg/dL)	48.52 ± 14.13	45.18 ± 12.71	**0.009**
T2DM, *n* (%)	56 (36.12)	235 (69.73)	**< 0.005**
HTN, *n* (%)	0	85 (25.22)	**< 0.005**
CVD, *n* (%)	0	47 (13.94)	**< 0.005**

Data with significant *p*-value (< 0.05) are shown in bold

*FBG* fasting blood glucose, *LDL* low density lipoprotein, *HDL* high density lipoprotein, *T2DM* type-2 diabetes mellitus, *HTN* hypertension, *CVD* cardiovascular disease

The allele frequencies of the SNPs rs1800592, rs10011540, rs3811791 (*UCP1*) and rs1805081 and rs1805082 (*NPC1*) are listed in Table 2 and genotype frequencies are mentioned in Additional file 1: Table S1. The mutant allele G of SNP rs1800592 on *UCP1* showed a significant association with obesity [OR, 1.52 (1.10–2.08); *p* = 0.009]. The distribution of all other SNPs in the patient and control population did not reveal any statistical significance. All genotype frequencies of the control group were consistent with Hardy-Weinberg equilibrium. Sequencing for Exon 2 and Exon 5 of *UCP1* revealed three genetic variants, Leu59Gln (1.51%), Ala64Thr (11.36%) and Met229Leu (14.39%) in the studied population and all were genotypically heterozygous. Therefore, these variants were excluded from the association analysis.

**Table 2:** Allelic distribution among patient and control cohort

Gene	SNP	Allele	Control (*n*)	Cases (*n*)	Model 1^a^			Model 2^b^			HWE
					OR	98.75%CI	*p*-value	OR	98.75%CI	*p* value	
UCP1	rs1800592	A	227	443	Ref						
		G	83	231	1.42	0.97–2.08	0.019	1.52	1.01–2.27	**0.009**	0.23
	rs10011540	T	283	627	Ref						
		G	27	47	0.78	0.42–1.47	0.338	0.78	0.40–1.52	0.361	0.85
	rs3811791	T	298	624	Ref						
		C	12	50	1.98	0.87–4.5	0.036	2.06	0.87–4.90	0.036	0.09
NPC1	rs1805081	T	275	605	Ref						
		C	35	69	0.89	0.51–1.55	0.617	0.84	0.47–1.52	0.487	0.43
	rs1805082	T	225	491	Ref						
		C	85	183	0.98	0.67–1.44	0.930	0.99	0.66–1.49	0.963	0.17

^a^ Unadjusted and ^b^Adjusted for Age, Gender and T2DM

Data with significant *p*-value (< 0.0125) are shown in bold

### Clinical, biochemical and genotypic characteristics of BMI stratified cohort

The association of rs1800592 and rs3811791 SNPs with obesity is arguable as results in different populations [10] and also in stratified obesity groups are controversial [24, 25]. To shed some light on the association, the patient group was further classified according to BMI namely moderate-obese and extreme-obese. The risk status of these cohorts was analyzed. The clinical and biochemical parameters of these cohorts are shown in Table 3. There were no significant differences among the biochemical parameters after stratification except for HDL which showed a significant difference in the extreme-obese cohort (*p* = 0.007). The allelic frequency distribution for *UCP1* and *NPC1* polymorphism was analyzed for the stratified cohorts, moderate-obese and extreme-obese (Table 4), similarly the frequencies of genotype in stratified cohort are given in Additional file 1: Table S2. The significant SNPs rs1800592 was not significantly associated with both stratified cohort, whereas another *UCP1* SNP rs3811791 was strongly associated with the moderate-obese (BMI 30–39.9 kg/m^2^) patients [OR = 2.89 (1.33–6.25); *p* = 0.007] but not with the extreme obese after stratification (Table 4).

**Table 3:** Clinical and biochemical parameters of study cohort after stratification into two groups based on their BMI

Clinical and biochemical parameters	Control (*n* = 155) (mean ± SD)	Moderate obese (*n* = 207) (mean ± SD)	*p*-value	Extreme obese (*n* = 130) (mean ± SD)	*p*-value
Age (years)	43.86 ± 14.54	50.45 ± 11.17	**< 0.005**	42.57 ± 13.72	0.248
Male / Female, *n* (%)	76 (49) / 79 (51)	91 (43.96) / 116 (56.03)	0.394	47 (36.15) / 83 (63.84)	**0.031**
BMI kg/m^2^	24.09 ± 2.60	34.15 ± 2.69	**< 0.005**	48.26 ± 11.94	**< 0.005**
FBG (mg/dL)	120.58 ± 56.75	161.33 ± 69.77	**< 0.005**	137.33 ± 72.41	**0.029**
Triglycerides (mg/dL)	100.00 ± 62.45	133.91 ± 66.23	**< 0.005**	141.54 ± 94.87	**< 0.005**
LDL (mg/dL)	115.25 ± 42.90	107.81 ± 37.11	0.078	117.62 ± 34.97	0.614
Cholesterol (mg/dL)	189.34 ± 134.62	176.86 ± 40.23	0.208	183.92 ± 41.72	0.659
HDL (mg/dL)	48.52 ± 14.13	45.78 ± 13.13	0.058	44.23 ± 12.00	**0.007**
T2DM, *n* (%)	56 (36.12)	175 (84.54)	**< 0.005**	60 (46.15)	0.092
HTN, *n* (%)	0	60 (28.98)	**< 0.005**	25 (19.23)	**< 0.005**
CVD, *n* (%)	0	34 (16.42)	**< 0.005**	13 (10)	**< 0.005**

Data with significant *p*-value (< 0.05) are shown in bold

*BMI* body mass index, *FBG* fasting blood glucose, *LDL* low density lipoprotein, *HDL* high density lipoprotein, *T2DM* type-2 diabetes mellitus, *HTN* hypertension, *CVD* cardiovascular disease

**Table 4:** Allelic distribution of patient population after stratifying to moderate-obese and extreme-obese groups based on their BMI

Gene	SNP	Allele	Control	Moderate obese	Model 1^a^			Model 2^b^			Extreme obese	Model 1^a^			Model 2^b^		
					OR	98.75%CI	*P* value	OR	98.75%CI	*p* value		OR	98.75%CI	*P* value	OR	98.75%CI	*p* value
UCP1	**rs1800592**	A	**227**	271	Ref						172	Ref					
		G	**83**	143	1.44	1.96–2.18	0.026	1.52	0.95–2.46	0.027	88	1.39	0.88–2.21	0.067	1.39	0.88–2.23	0.072
	**rs10011540**	T	**283**	382	Ref						245	Ref					
		G	**27**	32	0.87	0.44–1.73	0.633	1.01	0.46–2.23	0.967	15	0.64	0.28–1.48	0.183	0.58	0.25–1.37	0.113
	**rs3811791**	T	**298**	378	Ref						246	Ref					
		C	**12**	36	2.36	1.0–5.56	**0.011**	2.89	1.08–7.73	**0.007**	14	1.41	0.52–3.86	0.390	1.32	0.47–3.71	0.494
NPC1	**rs1805081**	T	**275**	371	Ref						234	Ref					
		C	**35**	43	0.91	0.50–1.66	0.698	0.78	0.39–1.56	0.376	26	0.87	0.44–1.73	0.619	0.86	0.43–1.75	0.612
	**rs1805082**	T	**225**	306	Ref						185	Ref					
		C	**85**	108	0.93	0.61–1.42	0.688	0.91	0.56–1.49	0.637	75	1.07	0.67–1.71	0.705	1.04	0.65–1.68	0.813

Data with significant *p*-value (< 0.0125) are shown in bold

^a^Unadjusted and ^b^Adjusted for Age, Gender and T2DM

### Associated *UCP1* SNPs (rs1800592 and rs3811791) vs biochemical parameters and co-existing diseases

**Fig. 1:**
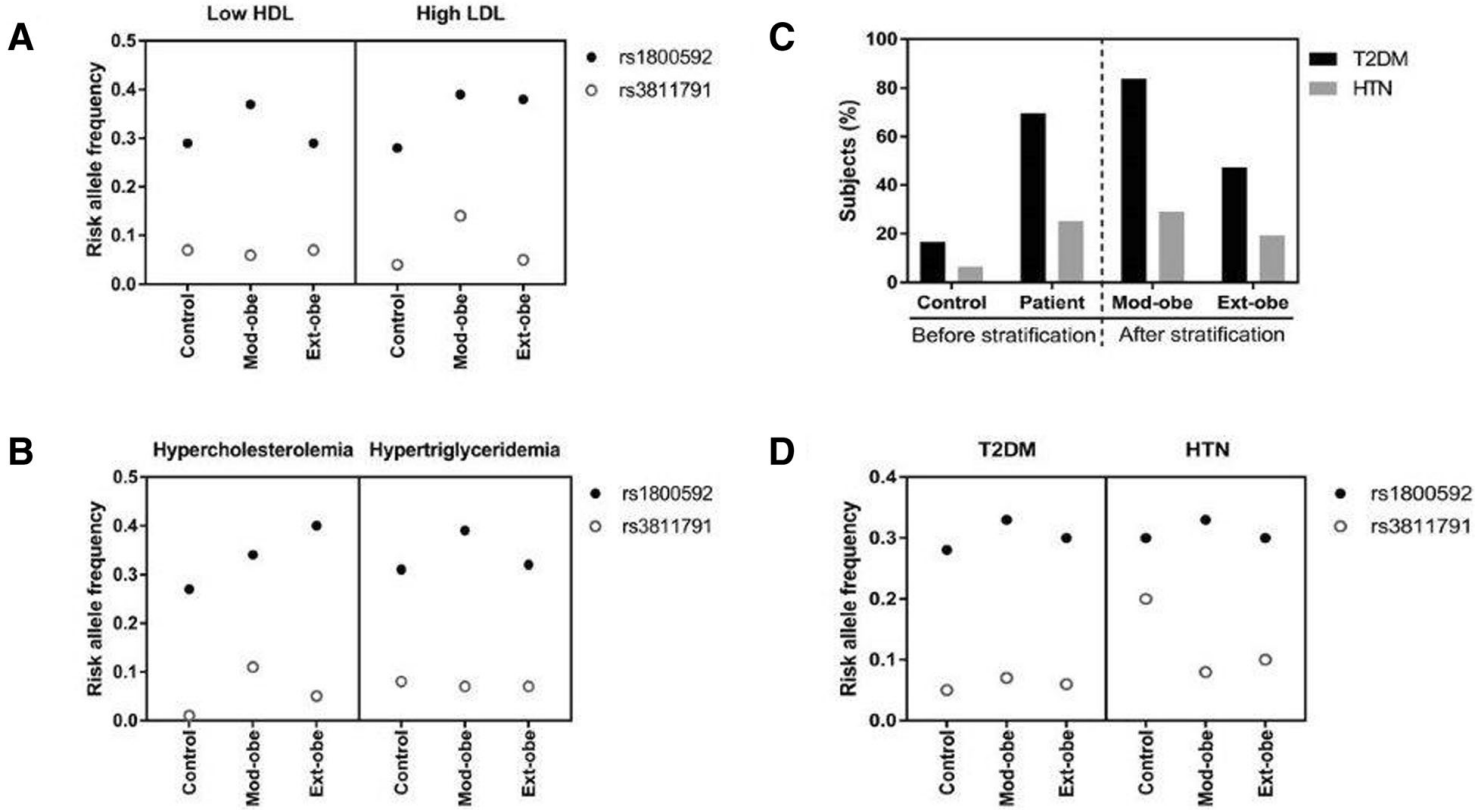
Frequency of the risk alleles of rs1800592 and rs3811791 in patients with abnormal HDL and LDL (**a**) and hypercholesterolemia and hypertriglyceridemia (**b**). Number of T2DM and HTN patients in the whole patient group and after the stratification (**c**); and the frequency of the risk alleles in these stratified cohort (**d**). Mod-obe: moderate-obese; Ext-obe: extreme-obese; T2DM: type-2 diabetes mellitus and HTN: hypertension

To verify the association of *UCP1* risk alleles (rs1800592 and rs3811791) with the abnormal biochemical parameters and co-existing diseases such as T2DM and HTN in the control, moderate-obese and extreme-obese cohorts, the risk allele frequencies (RAF) of both SNPs were calculated and plotted as shown in Fig. 1. The RAF of SNP rs1800592 was higher in the moderate-obese cohort with abnormal HDL and LDL levels compared to the control and extreme-obese cohorts. In rs3811791, the RAF was equally distributed to each cohort for low HDL level but for high LDL level, it was higher in the moderate-obese cohort (Fig. 1a). For hypercholesterolemia, the RAF of rs1800592 was higher in the extreme-obese cohort whereas, rs3811791 was higher in the moderate-obese. The RAF of rs1800592 was higher in the moderate-obese cohort for hypertriglyceridemia whereas, that of rs3811791 did not show any difference between these cohorts (Fig. 1b) (Additional file 1: Table S3).

T2DM (70%) and HTN (25.2%) were co-existing diseases in the total patient cohort. In 207 moderate-obese subjects, 84% had T2DM and 25% had HTN, whereas in the 130 extreme-obese subjects, 47.3% had T2DM and 19.2% had HTN (Fig. 1c). Allele frequency analysis showed that the RAF of rs1800592 was higher in the moderate-obese cohort in both T2DM and HTN patients, whereas the other risk allele of rs3811791 was not remarkably changed after stratification (Fig. 1d).

### Associated *UCP1* SNPs (rs1800592 and rs3811791) vs age and gender with BMI

**Fig. 2:**
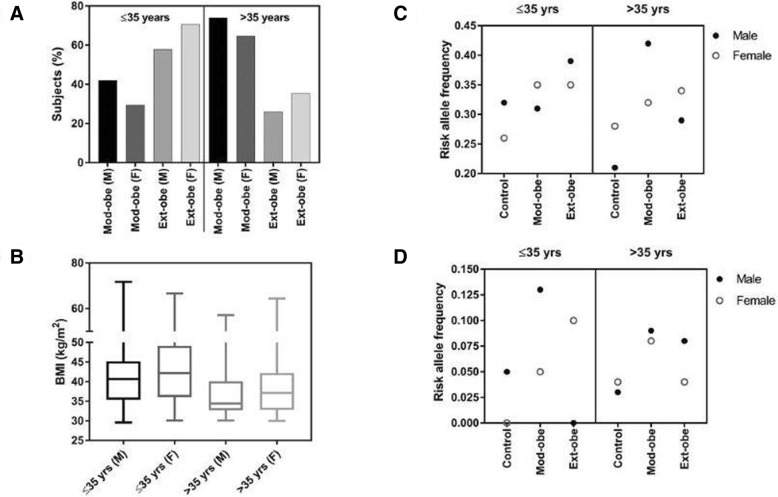
Percentage of subjects after subdividing the total patient cohort based on their age, sex and BMI (**a**); Box plot representing the association of BMI with these subdivided groups (**b**); Frequency of risk alleles in these subdivided groups: risk allele frequency of rs1800592 (**c**) and risk allele frequency of rs3811791 (**d**). Mod-obe (M): moderate-obese male, Mod-obe (F): moderate-obese female, Ext-obe (M): extreme-obese male and Ext-obe (F): extreme-obese female

It has been reported that the prevalence of obesity is higher in young females in the Saudi population than males. In our study, it was found that the majority of the subjects were female in both stratified groups. To confirm if there was any association of age and gender with BMI, the patient population was subdivided based on age (≤35 years and > 35 years) and gender. We observed a higher number of young females (≤35 years) in the extreme-obese cohort whereas, the males > 35 years of age dominated the moderate-obese cohort (Fig. 2a). To determine the association of age and gender with BMI, a box plot with BMI on Y-axis and subdivided patient groups on X-axis was plotted as shown in Fig. 2b. In both age groups, the increased BMI was associated with female subjects (≤35 years: mean BMI = 43.26 ± 8.58 kg/m^2^; and > 35 years: mean BMI = 38.64 ± 7.27 kg/m^2^).

To verify whether these two risk alleles of *UCP1* had any association with age and gender, the RAF was studied after subdividing the patient group based on age and gender. The RAF of rs1800592 was higher in the extreme-obese males in ≤35 years sub group whereas, in the > 35 subgroup, the RAF in the moderate-obese males was high (Fig. 2c). For rs3811791, the RAF was higher in females aged ≤35 years with extreme-obesity and in males aged ≤35 years with moderate-obesity. In the > 35 years group, the RAF was high in both male and female in the moderate-obese cohort (Fig. 2d).

## Discussion

This study reports the association of common *UCP1* polymorphisms in an obese population in Saudi Arabia. The *UCP1* gene is considered to be a candidate gene for obesity and T2DM as the polymorphism of this gene reduces the mitochondrial membrane potential and mediates proton leak [26]. Mutations in these genes reduce the availability of functional proteins, which in turn, could reduce energy expenditure by increasing coupling of oxidative phosphorylation, thereby contributing to the development of obesity.

We selected the most studied polymorphisms of the *UCP1* gene with respect to obesity. Many studies have reported that polymorphisms (rs1800592, rs10011540, rs3811791) of the *UCP1* promoter region and rs45539933 and rs2270565 of the *UCP1* coding region are associated with obesity and T2DM. Among these polymorphisms, rs1800592 was the most studied polymorphism and the results were highly controversial in different populations [10]. To the best of our knowledge, no previous studies have reported an association of rs1800592 polymorphism from our geographic region. The importance of rs1800592 polymorphism in regulating the expression of the *UCP1* gene has previously been reported in obese subjects [27]. The presence of rs1800592 polymorphism in the *UCP1* gene was first identified in 1994 in a pilot study conducted on 261 Canadian patients and was associated with obesity and weight gain [28, 29]. Subsequently, several studies reported the status of this polymorphism with obesity and other associated parameters in different populations, but it still remains arguable as it exhibits different allele frequencies in various ethnic populations.

In this study, we found that *UCP1* gene polymorphism rs1800592 is significantly associated with increased BMI. When the patient cohort was stratified based on their BMI, other UCP1 SNP, rs3811791, was associated with moderate-obese patients. Several independent studies conducted in different ethnicities supported the association between the G-allele of rs1800592 and obesity, BMI or other obesity-related parameters [30–32]. On the other hand, a number of studies have reported a lack of association of rs1800592 with an obese population with different ethnic background [33–37]. Previously, the association of another significant *UCP1* SNP rs3811791, was reported in Japanese and Indian diabetic patients [13, 38]. For the first time, this study reports the association of rs3811791 SNP with obesity, specifically in moderate-obese patients. We could not determine any association of other *UCP1* polymorphisms, namely rs10011540, rs45539933 and rs2270565 in this population. The association of *NPC1* polymorphisms rs1805081 and rs1805082 has been reported in European subjects [19]. However, in a study conducted on obese Chinese children, rs1805081 was not significantly associated [20]. Obesity in the population included in the current study was not associated with the reported *NPC1* polymorphisms, which is in accordance with an earlier study conducted in Saudi Arabia, indicating that this SNP is neither associated with obesity nor BMI [39].

Several studies have reported that the G-allele of rs1800592 is associated with a low level of HDL [40], and high level of triglyceride [41] and LDL [42] in obese subjects in different populations. Similarly, in this study, we observed an increased RAF (G-allele) with lower HDL and higher LDL and hypertriglyceridemia in the moderate-obese cohort than in the extreme-obese and control cohorts. These observations reflect the effective involvement of UCP1-mediated pathways in the regulation of obesity-related metabolic parameters in moderate-obese subjects. However, in extreme-obese cases, other functional pathways are effectively involved which may increase BMI, thereby increasing the risk of metabolic complications.

*UCP1* is predominantly expressed in brown adipose tissue and eminently participates in the process of thermogenesis [43, 44]. Recent studies conducted in animal models using targeted chemical uncouplers and adipose tissue- and skeletal muscle-targeted overexpression of UCP1 resulted in decreased hypertriglyceridemia, glucose homeostasis by increased insulin sensitivity and glucose uptake and as well as a decreased level of diet- and genetic-induced obesity [44–48]. There was a significant number of diabetic patients in both moderate-obese (four-fold increase) and extreme-obese cohorts (two-fold increase) compared to the control cohort. The increased number of T2DM patients and increased RAF of rs1800592 in the moderate-obese cohort sheds light on the association of this SNP with obesity associated with T2DM. The association of rs1800592 with T2DM is controversial as the studies conducted in different ethnicities exhibited varying results [31, 33, 35, 49]. *UCP1* may play a major role in inducing insulin-resistance and diabetes in moderate-obese cases. This observation may also help to accelerate the investigation on how these two complicated conditions, obesity and T2DM, are inter-related with each other.

**Fig. 3:**
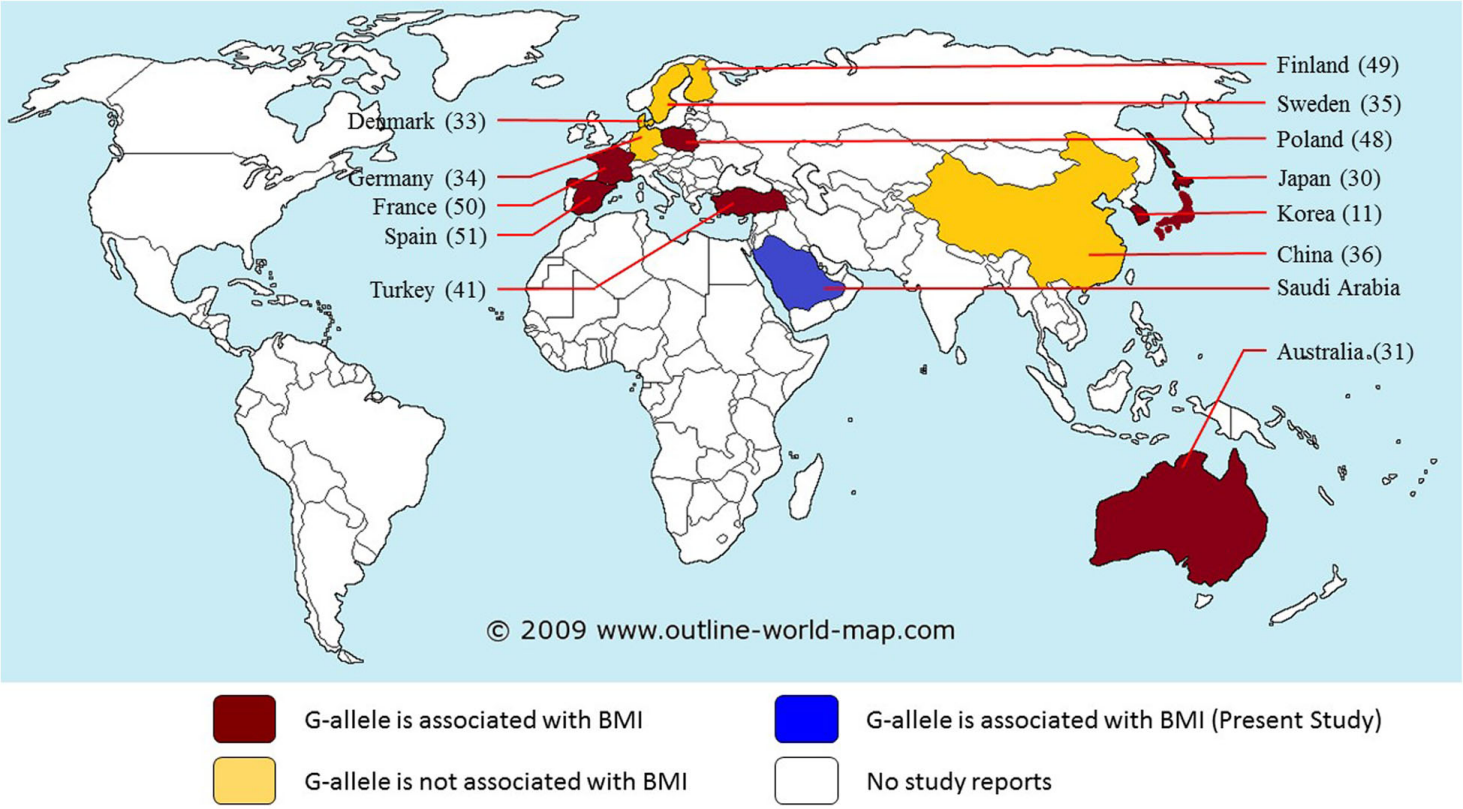
Schematic diagram represents how the risk allele G of rs1800592 is distributed in different population worldwide

A schematic representation of the available data which specifically reported how this rs1800592 risk allele (allele G) is associated with obeisty worldwide is shown in Fig. 3 [11, 30, 31, 33–36, 41, 48–51]. A recent population-based study reported that BMI levels were increasing in the Saudi population, with a more rapid increase in females than males [4]. Similarly, Memish et al. (2014) reported that the level of obesity in Saudi females was higher than that in males (33.5% vs 24.1%) [5]. The present study revealed a higher ratio of young obese female patients within the extreme-obese cohort compared to the moderate-obese cohort.

## Conclusion

The present study reveals a significant association of rs1800592 and rs3811791 polymorphisms in the promoter region of the *UCP1* gene with obese population in Saudi Arabia. The associated *UCP1* polymorphisms in the moderate-obese group may regulate impaired energy metabolism which plays a significant role in the initial stages of obesity. *NPC1* polymorphisms were not found to be an important risk factor for obesity in Saudi Arabia.

## Additional file

Additional file 1: **Table S1.** Genotypic distribution among patient and control cohort. Genotypic odds ratio for all cases and controls, unadjusted and adjusted for Age, Sex and T2D. **Table S2.** Genotypic distribution among stratified cohort. Genotypic odds ratio among patient population stratified for BMI; moderate-obese and extreme-obese groups. **Table S3.** Distribution of risk alleles in normal and abnormal levels of biochemical parameters. Association of the risk alleles frs1800592 and rs3811791 with HDL, LDL, Triglycerides and total cholesterol. (DOCX 42 kb)

## Abbreviations

BMI: Body mass index
EDTA: Ethylenediamine tetra acetic acid
ELISA: Enzyme-linked immunosorbent assay
HDL: High-density lipoprotein
LDL: Low-density lipoprotein
LE/LY: Late endosome/lysosome
NPC1: Niemann-Pick C1
PCR: Polymerase Chain Reaction
SNP: Single Nucleotide Polymorphism
T2DM: Type 2 diabetes mellitus
UCP1: Uncoupling protein 1
