# Supplementary material for: Association of Uncoupling Protein 1 (UCP1) gene polymorphism with obesity: a case-control study
Source: BMC Med Genet. 2018 Nov 20;19:203. doi: 10.1186/s12881-018-0715-5 (PMC6247512; doi:10.1186/s12881-018-0715-5)
Supplement: Supplementary file 1 — Table S1. Genotypic distribution among patient and control cohort. Genotypic odds ratio for all cases and controls, unadjusted and adjusted for Age, Sex and T2D. Table S2. Genotypic distribution among stratified cohort. Genotypic odds ratio among patient population stratified for BMI; moderate-obese and extreme-obese groups. Table S3. Distribution of risk alleles in normal and abnormal levels of biochemical parameters. Association of the risk alleles frs1800592 and rs3811791 with HDL, LDL, Triglycerides and total cholesterol. (DOCX 42 kb) [file 12881_2018_715_MOESM1_ESM.docx]

**Table S1** Genotypic distribution among patient and control cohort.

| **Gene** | **SNP** | **Genotype** | **Control** | **Cases** | **Model 1^*^** | | | **Model 2^#^** | | |
| --- | --- | --- | --- | --- | --- | --- | --- | --- | --- | --- |
|  |  |  |  |  | **OR** | **98.75%CI** | **p value** | **OR** | **98.75%CI** | **p value** |
| UCP1 | rs1800592 | TT | 86 | 154 | Ref |  |  |  |  |  |
|  |  | TC | 55 | 135 | 1.37 | 0.81-2.31 | 0.13 | 1.43 | 0.82-2.49 | 0.11 |
|  |  | CC | 14 | 48 | 1.91 | 0.83-4.40 | 0.05 | 2.18 | 0.92-5.57 | 0.03 |
|  |  | TC+CC | 69 | 183 | 1.48 | 0.91-2.41 | 0.04 | 1.57 | 0.94-2.65 | 0.03 |
|  | rs10011540 | TT | 129 | 294 | Ref |  |  |  |  |  |
|  |  | TG | 25 | 39 | 0.68 | 0.34-1.37 | 0.17 | 0.78 | 0.38-1.64 | 0.42 |
|  |  | GG | 1 | 4 | 1.75 | 0.10-29.0 | 0.62 | 0.86 | 0.05-14.54 | 0.90 |
|  |  | TG+GG | 26 | 43 | 0.72 | 0.37-1.42 | 0.23 | 0.79 | 0.38-1.62 | 0.43 |
|  | rs3811791 | TT | 144 | 291 | Ref |  |  |  |  |  |
|  |  | TC | 10 | 42 | 2.07 | 0.83-5.20 | 0.04 | 1.9 | 0.73-4.98 | 0.09 |
|  |  | CC | 1 | 4 | 1.97 | 0.12-32.68 | 0.54 | 3.45 | 0.17-60.69 | 0.29 |
|  |  | TC+CC | 11 | 46 | 2.06 | 0.86-4.96 | 0.04 | 2.02 | 0.80-5.07 | 0.05 |
| NPC1 | rs1805081 | TT | 121 | 269 | Ref |  |  |  |  |  |
|  |  | TC | 33 | 67 | 0.91 | 0.50-1.66 | 0.70 | 0.88 | 0.47-1.67 | 0.63 |
|  |  | CC | 1 | 1 | 0.44 | 0.01-15.54 | 0.57 | 0.56 | 0.01-26.64 | 0.70 |
|  |  | TC+CC | 34 | 68 | 0.89 | 0.50-1.62 | 0.65 | 0.87 | 0.47-1.64 | 0.60 |
|  | rs1805082 | TT | 85 | 179 | Ref |  |  |  |  |  |
|  |  | TC | 55 | 133 | 1.14 | 0.69-1.92 | 0.50 | 1.15 | 0.66-2.0 | 0.52 |
|  |  | CC | 15 | 25 | 0.79 | 0.33-1.90 | 0.51 | 0.84 | 0.34-2.14 | 0.65 |
|  |  | TC+CC | 70 | 158 | 1.07 | 0.66-1.74 | 0.72 | 1.08 | 0.65-1.82 | 0.69 |

^*^ Unadjusted and ^#^Adjusted for Age, Gender and T2DM

**Table S2** Genotypic distribution among stratified cohort.

| **Gene** | **SNP** | **Genotype** | **Control** | **Moderate Obese** | **Model 1^*^** | | | **Model 2^#^** | | | **Extreme Obese** | **Model 1^*^** | | | **Model 2^#^** | | |
| --- | --- | --- | --- | --- | --- | --- | --- | --- | --- | --- | --- | --- | --- | --- | --- | --- | --- |
|  |  |  |  |  | **OR** | **98.75%CI** | **p value** | **OR** | **98.75%CI** | **p value** |  | **OR** | **98.75%CI** | **p value** | **OR** | **98.75%CI** | **p value** |
| UCP1 | rs1800592 | TT | 86 | 96 | Ref |  |  |  |  |  | 58 | Ref |  |  |  |  |  |
|  |  | TC | 55 | 79 | 1.28 | 0.72-2.29 | 0.27 | 1.18 | 0.60-2.30 | 0.51 | 56 | 1.51 | 0.80-2.85 | 0.10 | 1.54 | 0.80-2.93 | 0.10 |
|  |  | CC | 14 | 32 | 2.04 | 0.84-4.94 | 0.04 | 2.55 | 0.88-7.42 | 0.02 | 16 | 1.69 | 0.62-4.64 | 0.20 | 1.66 | 0.60-4.91 | 0.22 |
|  |  | TC+CC | 69 | 111 | 1.44 | 0.84-2.45 | 0.09 | 1.42 | 0.77-2.64 | 0.15 | 72 | 1.54 | 0.85-2.81 | 0.07 | 1.56 | 0.85-2.88 | 0.06 |
|  | rs10011540 | TT | 129 | 179 | Ref |  |  |  |  |  | 115 | Ref |  |  |  |  |  |
|  |  | TG | 25 | 24 | 0.69 | 0.32-1.50 | 0.23 | 1.05 | 0.43-2.61 | 0.87 | 15 | 0.67 | 0.28-1.61 | 0.26 | 0.62 | 0.25-1.54 | 0.20 |
|  |  | GG | 1 | 4 | 2.88 | 0.17-47.75 | 0.35 | 1.2 | 0.64-18.72 | 0.87 | 0 | - | - | - | - | - | - |
|  |  | TG+GG | 26 | 28 | 0.77 | 0.37-1.62 | 0.4 | 1.07 | 0.45-2.54 | 0.84 | 15 | 0.64 | 0.27-1.54 | 0.21 | 0.59 | 0.24-1.45 | 0.15 |
|  | rs3811791 | TT | 144 | 175 | Ref |  |  |  |  |  | 116 | Ref |  |  |  |  |  |
|  |  | TC | 10 | 28 | 2.3 | 0.88-6.03 | 0.03 | 2.15 | 0.71-6.61 | 0.08 | 14 | 1.74 | 0.59-5.11 | 0.20 | 1.73 | 0.57-5.23 | 0.21 |
|  |  | CC | 1 | 4 | 3.29 | 0.20-54.48 | 0.29 | 9.11 | 0.42-178.33 | 0.07 | 0 | - | - | - | - | - | - |
|  |  | TC+CC | 11 | 32 | 2.4 | 0.95-5.98 | 0.02 | 2.59 | 0.90-7.44 | 0.02 | 14 | 1.58 | 0.55-4.53 | 0.28 | 1.53 | 0.52-4.48 | 0.325 |
| NPC1 | rs1805081 | TT | 121 | 164 | Ref |  |  |  |  |  | 105 | Ref |  |  |  |  |  |
|  |  | TC | 33 | 43 | 0.96 | 0.50-1.84 | 0.88 | 0.93 | 0.44-1.98 | 0.81 | 24 | 0.84 | 0.39-1.77 | 0.55 | 0.80 | 0.37-1.73 | 0.48 |
|  |  | CC | 1 | 0 | - | - | - | - | - | - | 1 | 1.15 | 0.03-40.03 | 0.92 | 1.56 | 0.04-57.23 | 0.76 |
|  |  | TC+CC | 34 | 43 | 0.93 | 0.48-1.78 | 0.79 | 0.88 | 0.42-1.87 | 0.69 | 25 | 0.84 | 0.40-1.77 | 0.57 | 0.82 | 0.38-1.75 | 0.52 |
|  | rs1805082 | TT | 85 | 111 | Ref |  |  |  |  |  | 68 | Ref |  |  |  |  |  |
|  |  | TC | 55 | 84 | 1.17 | 0.66-2.05 | 0.49 | 1.26 | 0.65-2.43 | 0.38 | 49 | 1.11 | 0.58-2.10 | 0.67 | 1.05 | 0.55-2.04 | 0.83 |
|  |  | CC | 15 | 12 | 0.61 | 0.22-1.72 | 0.23 | 0.54 | 0.17-1.82 | 0.20 | 13 | 1.08 | 0.38-3.03 | 0.85 | 1.07 | 0.37-3.03 | 0.87 |
|  |  | TC+CC | 70 | 96 | 1.05 | 0.61-1.79 | 0.81 | 1.09 | 0.67-1.77 | 0.72 | 62 | 1.11 | 0.61-2.00 | 0.67 | 1.06 | 0.57-1.95 | 0.80 |

^*^ Unadjusted and ^#^Adjusted for Age, Gender and T2DM

**Table S3** Distribution of risk alleles in normal and abnormal levels of biochemical parameters.

|  |  | **rs1800592 risk allele** | | **p value** | **rs3811791 risk allele** | | **p value** |
| --- | --- | --- | --- | --- | --- | --- | --- |
|  |  | **Normal** | **Abnormal** |  | **Normal** | **Abnormal** |  |
| **HDL** | Control | 52 | 27 | Ref | 4 | 6 | Ref |
|  | Moderate | 83 | 52 | 0.53 | 24 | 10 | 0.08 |
|  | Extreme | 48 | 26 | 0.90 | 8 | 6 | 0.41 |
| **LDL** | Control | 55 | 26 | Ref | 8 | 4 | Ref |
|  | Moderate | 99 | 36 | 0.39 | 21 | 13 | 0.76 |
|  | Extreme | 52 | 36 | 0.24 | 9 | 5 | 0.90 |
| **Triglyceride** | Control | 71 | 8 | Ref | 8 | 2 | Ref |
|  | Moderate | 114 | 21 | 0.26 | 30 | 4 | 0.50 |
|  | Extreme | 78 | 9 | 0.96 | 12 | 2 | 0.71 |
| **Cholesterol** | Control | 56 | 23 | Ref | 9 | 1 | Ref |
|  | Moderate | 96 | 36 | 0.77 | 22 | 12 | 0.12 |
|  | Extreme | 54 | 33 | 0.23 | 10 | 4 | 0.27 |
